# A convolutional recurrent neural network with attention framework for speech separation in monaural recordings

**DOI:** 10.1038/s41598-020-80713-3

**Published:** 2021-01-14

**Authors:** Chao Sun, Min Zhang, Ruijuan Wu, Junhong Lu, Guo Xian, Qin Yu, Xiaofeng Gong, Ruisen Luo

**Affiliations:** 1 College of Electrical Engineering, Sichuan University, Chengdu, 610065 China; 2 Institute of Urban and Rural Planning and Design Zhejiang, Hangzhou, 310007 China; 3 Chengdu Dagongbochuang Information Technology Co., Ltd., Chengdu, 610059 China

**Keywords:** Engineering, Electrical and electronic engineering

## Abstract

Most speech separation studies in monaural channel use only a single type of network, and the separation effect is typically not satisfactory, posing difficulties for high quality speech separation. In this study, we propose a convolutional recurrent neural network with an attention (CRNN-A) framework for speech separation, fusing advantages of two networks together. The proposed separation framework uses a convolutional neural network (CNN) as the front-end of a recurrent neural network (RNN), alleviating the problem that a sole RNN cannot effectively learn the necessary features. This framework makes use of the translation invariance provided by CNN to extract information without modifying the original signals. Within the supplemented CNN, two different convolution kernels are designed to capture information in both the time and frequency domains of the input spectrogram. After concatenating the time-domain and the frequency-domain feature maps, the feature information of speech is exploited through consecutive convolutional layers. Finally, the feature map learned from the front-end CNN is combined with the original spectrogram and is sent to the back-end RNN. Further, the attention mechanism is further incorporated, focusing on the relationship among different feature maps. The effectiveness of the proposed method is evaluated on the standard dataset MIR-1K and the results prove that the proposed method outperforms the baseline RNN and other popular speech separation methods, in terms of GNSDR (gloabl normalised source-to-distortion ratio), GSIR (global source-to-interferences ratio), and GSAR (gloabl source-to-artifacts ratio). In summary, the proposed CRNN-A framework can effectively combine the advantages of CNN and RNN, and further optimise the separation performance via the attention mechanism. The proposed framework can shed a new light on speech separation, speech enhancement, and other related fields.

## Introduction

The purpose of speech separation is to separate the target speech from the background interference^1–4^, also known as the “cocktail party problem”. Depending on the number of sensors or microphones, separation methods can be divided into single channel methods (single microphone) and array methods (multiple microphones). The sound collected by the microphone might include noises, accompaniments and other interference items, hence, the accuracy of the speech recognition might not be satisfactory without speech separation^5,6^. Therefore, speech separation is of great value in the area of signal processing, such as speaker recognition and automatic speech recognition.

General signals, such as songs, have a mixture of both vocals and accompaniment. The research content of this paper focuses on the separation of singing voice from monaural recordings, which is a basic and important branch in speech separation.

The method of speech separation can be divided into two branches: traditional separation based on statistical features and current separation based on deep learning. Huang et al.^7^ used robust principal component analysis (RPCA) to separate the singing voice and the accompaniment. Yang et al.^8^ considered the singing voice to be a sparse signal, while the accompaniment part can be represented by a low rank. However, this is only an idealised assumption. Yang et al.^9^ held the assumption that accompaniment in reality is not always of low rank; thus, a new algorithm called Multiple Low Rank Representation (MLRR) was proposed. MLRR decomposed the speech signal magnitude spectrogram into two low-rank matrices, improved in GNSDR and GSIR indicators. Since most methods rely on the signal pitch to solve this separation task, the key difficulties are a consequence of the incorrect judgment of the signal pitch or the failure to recognise the pure singing voice. Thus, Zhang et al.^10^ proposed a new algorithm based on latent component analysis of time-frequency representation for separation and achieved a good separation effect. Roux et al.^11^ proposed a deep non-negative matrix factorisation (DNMF) separation algorithm and expanded the NMF iteration, improving the accuracy with fewer parameters.

In the recent years, deep learning has been advantageous in the fields of natural language processing (NLP). Wang et al.^12^ used deep neural networks (DNNs) to learn the ideal binary mask, and treated source separation problems as binary classification problems. Similarly, Uhlich et al.^13^ extracted an instrument signal from music by using DNNs. Nugraha et al.^14^ used DNNs to learn the spectral features of the signal source, and used Wiener filters to distinguish between signals and noise. Owing to the fact that a speech signal is represented as one-dimensional time series data having long-short-term dependence, Huang et al.^15^ applied RNN to speech separation to learn the information of previous time steps and obtain long context information. Uhlich et al.^16^ used data augmentation, and integrated different networks to separate the music sources. Sebastian et al.^17^ used the modified group delay (MOD-GD) function to learn the time-frequency mask of the source.

These days, CNNs are the most representative networks for the two-dimensional image processing. One-dimensional time series speech signals can be converted into two-dimensional images through time-frequency conversion algorithms^18,19^, such as short-time Fourier transform (STFT). In this way, CNNs have also been successfully applied to speech separation^20,21^. Ronneberger et al.^22^ designed a U-net network for biomedical image segmentation. Jansson et al.^23^ used U-net’s advantages for image segmentation, successfully migrated its framework to speech separation, and provided a creative idea for speech separation. Aiming at the problem that most separation networks often ignore, the phase information of a speech signal, which can make the separation performance dependent on the hyperparameters, Stoller et al.^24^ proposed a new network called Wave-U-net that can make the U-net adapt to one-dimensional time-domain information by utilising the unique phase information of the speech signal. Naithani et al.^25^ proposed a combination of CNN and Long Short-Term Memory (LSTM) to solve the problem of source separation for a single channel signal. Yuan et al.^26^ proposed the Enhanced Feature Network (EFN), which has achieved a certain improvement in both GNSDR and GSAR indicators compared to the DRNN.

We noticed that the information of the speech signal has complex time correlation, and that the speech signal between different timeframes may have semantic correlation. RNN, which can model sequential data, such as text, speech, etc. is the most commonly used neural network in time-domain processing. However, its ability to perform feature learning is insufficient. We believe CNN, which has obvious advantages in image processing, can make up for this defect. Therefore, we introduce a CNN as the front-end of an RNN in order to extract global features and fine details of speech spectrograms, such as harmonics. The back-end still uses an RNN which has a “memory” function for sequence data.

**Figure 1:**
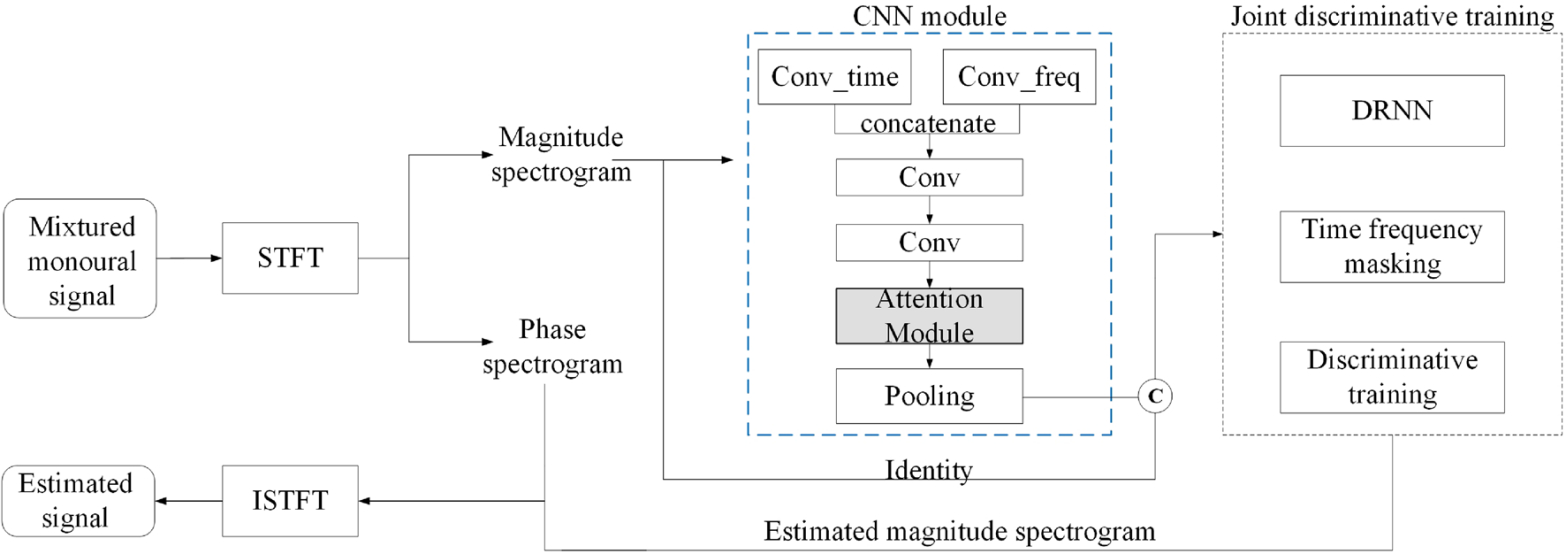
Overview of the proposed CRNN-A framework for speech separation.

We also noticed that the various prior studies on separation rarely pay much attention to the dependence between different feature maps. Referring to Hu et al.^27^ who successfully applied the attention mechanism in image classification, we also add attention to our speech separation task. The overall separation framework is shown in Fig. 1. Firstly, the mixed monaural source signal is transferred from the time-domain to the frequency-domain through STFT, and the obtained magnitude spectrogram is used as the input for the front-end CNN. Whereas Li et al.^28^ verified that the combination of different shapes of convolution kernels can effectively extract the speech feature information in the task of speech emotion recognition (SER), and we adapted it to speech separation. Two different convolution kernels were used to extract the time-domain and frequency-domain features of the spectrogram, respectively (Convtime and Convfreq). After concatenating their respective time-domain feature maps with frequency-domain feature maps, they then go through a series of convolutional and pooling operations to learn the deep level speech features and reduce the parameters, respectively. Finally, the feature map obtained from the pooling layer is combined with the original magnitude spectrogram. These are taken together as the input of the back-end RNN to separate the singing voice from the accompaniment. Where Conv_time represents the convolution in the time-domain, Conv_freq represents the convolution in the frequency-domain, and ©represents the concatenate in the frequency-domain.

## Methods

### Signal preprocessing (STFT)

Since the speech signal is one-dimensional, it must be converted into a two-dimensional image before using the CNN to train and learn its features. The best way is to use the STFT to convert it into magnitude spectrogram. As shown in Table 1, when the one-dimensional time-domain signal is converted to the frequency-domain signal, the size of the obtained input spectrogram is 513 $\times$ 10, where 513 represents the frequency point and 10 represents the time frame.

**Table 1:** Parameter settings of the entire network.

Structure		Parameter	Value
STFT		Sample window size	1024
		Sample hop_length	256
		Sample rate	16,000 Hz
		Input spectrogram	513 $\times$ 10
CNN		Conv kernel size (time-domain)	10 $\times$ 2
		Conv kernel size (frequency-domain)	2 $\times$ 10
		Conv kernel size (other convolutional layers)	2 $\times$ 2
		Conv stride	1 $\times$ 1
		Activation function	leaky$\_$relu
		Pool kernel size	2x1
		Pool stride	2x1
RNN		Number of layers of RNN	3
		Number of hidden layer neurons	1024
		Activation function	ReLU
Training parameters		Learning rate	1e−4
		Optimiser	Adam^42^
		Iterations	2e+4
		Batch size	64

### Front-end structure (CNN)

**Figure 2:**
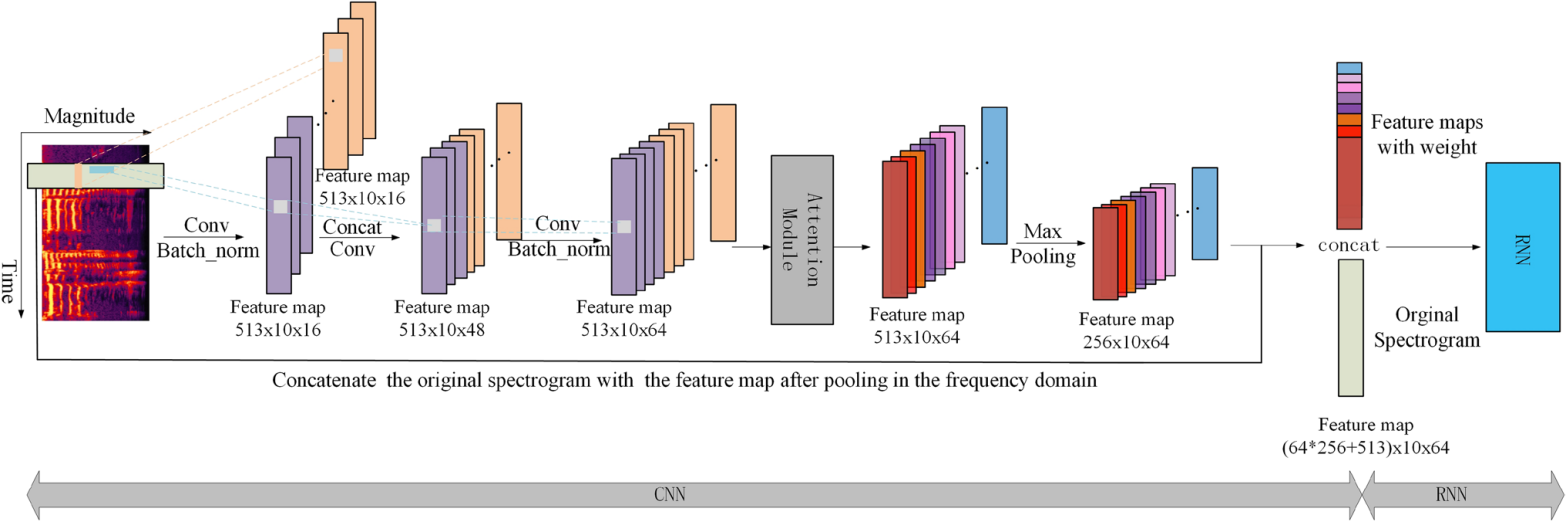
Structure of the proposed CRNN-A framework.

Because this paper deals with speech signals, the general square convolution kernels (such as 3 $\times$ 3 kernel) cannot make good use of the speech time-frequency domain feature information. Therefore, the two sets of convolution kernels in this paper are rectangular-shaped kernels (2 $\times$ 10, 10 $\times$ 2), which can capture the time-domain and frequency-domain contextual information from the input spectrogram, respectively. The padding mode of convolution layers is set as ‘SAME’; therefore, the size of the feature map does not change its size after convolution. As shown in Fig. 2, after convolution in the time-domain and frequency-domain, the size of the two feature maps is the same; both are 513 $\times$ 10 $\times$ 16, where 16 represents the number of feature maps. We concatenate the two types of feature maps to obtain a larger map (513 $\times$ 10 $\times$ 32). Using this method to concatenate the feature maps extracted by convolution kernels of different shapes can be regarded as encoding different feature information. And after concatenating these two, the feature maps went through two consecutive additional convolutional layers, and the number of feature maps were 48 and 64, respectively (513 $\times$ 10 $\times$ 48, 513 $\times$ 10 $\times$ 64).

### Front-end structure (attention module)

**Figure 3:**
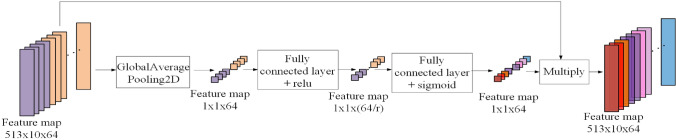
Attention mechanism in the separation framework^27^.

After the last layer of the convolution operation, we added the attention mechanism^27^. The purpose is to make each feature map correspond to a weight, and reduce the weights of those feature maps which do not contribute much to the separation, or are redundant. Simultaneously, it can highlight the useful feature maps. In general, it makes the feature maps more distinguishable. As shown in Fig. 3, as the feature map of the last convolutional layer first passes a global average pooling layer, the size of the spectrogram is reduced to 1 $\times$ 1, and is then linearly mapped through a fully connected layer with an activation function of ReLU. At this time, the dimension of the spectrogram is 1 $\times$ 1 $\times$ (64/r). Where r is a hyperparameter, which represents the reduction ratio, the function of which is to reduce the number of network parameters. Next, through a second fully connected layer with an activation function of leaky$\_$relu, the number of feature maps is restored to the previous number (1 $\times$ 1 $\times$ 64). Finally, we multiply the output of the second fully connected layer with the original last layer of the convolutional operation, restoring the resolution of the spectrogram to 512 $\times$ 10. Modelling the relationship between different feature maps in this way can simplify the network’s training process and enhance the network’s generalisation ability. Each convolutional layer is processed by batch normalisation (BN)^29^ to speed up the training process of the network. The chosen activation function is ReLU.

### Front-end structure (Pooling layer)

In order to compress the number of network parameters, the pooling layer is used to reduce the dimensions of the feature maps after the attention layer, which in turn can reduce overfitting of the network and improve its ability to generalise. Inspired by the references^25^, we set the pooling kernel size to (2 $\times$ 1), such that the time dimension is not changed but the frequency dimension is halved. Finally, we concatenate the original spectrogram with the spectrogram output of the pooling layer in the frequency-domain and use it as input to the back-end RNN. Concatenating different feature maps (the original spectrogram and the feature map after pooling) in the frequency domain can also be regarded as exploiting and fusing together features.

### Back-end structure (RNN)^15^

**Figure 4:**
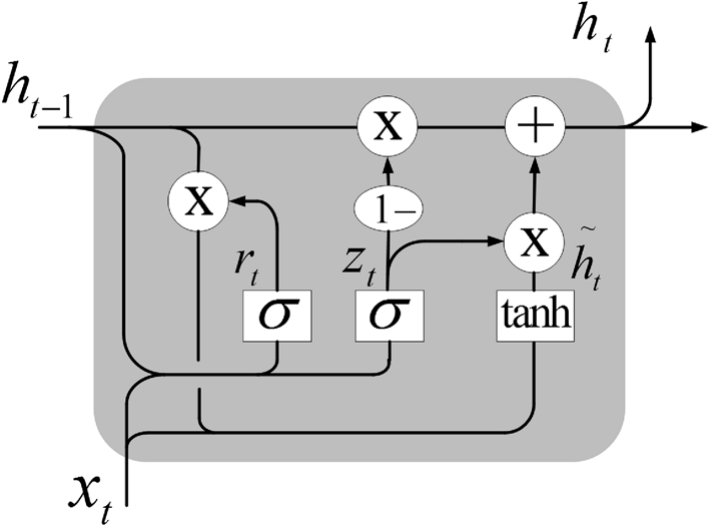
The specific structure of GRU^34^.

The function of RNN is mainly to use the feature information learned by the front-end to separate singing voice and accompaniment. Currently two variants are commonly used: LSTM^30,31^ and GRU^32^. Weninger et al.^33^ used LSTM to perform speech separation in a single channel. GRU is a variant of LSTM, which was proposed by Cho et al.^34^ It mainly combines the forget gate and input gate into a single update gate. The latter model is simpler than the standard LSTM model. Its effect is similar to LSTM, but the parameters are reduced, such that it is not easy to overfit. The variant of RNN used in this paper is GRU (Fig. 4).

The two gates of GRU are the reset gate ${r}_{t}$ and the update gate ${z}_{t}$. The reset gate ${r}_{t}$ is used to determine how much of the previous memory information needs to be retained. The smaller ${r}_{t}$ is, the lesser information from the previous state is written. The update gate ${z}_{t}$ is used to control the extent to which the state information from the previous moment is brought into the current state. The larger ${z}_{t}$ is, the more state information from the previous moment is brought in. Here ${x}_{t}$ represents the speech feature map of the mixture signal learned by the front-end CNN. ${h}_{t}$ represents the output speech feature information of the RNN hidden layer at the current time *t*, and ${h}_{t-1}$ represents the speech feature information output by the RNN hidden layer at the previous time ${t-1}$. Here [] means that the two vectors are connected, is a mathematical shorthand. We can expand it to get the equation on the right. And * implies matrix multiplication, ${W}_{r}$, ${W}_{z}$ and *W* are weight matrices in the neural network.

1 $$\begin{aligned} r_{t}= & {} \sigma (W_{r} \cdot [h_{t-1},x_{t}]) = \sigma (x_{t} W_{xr} + h_{t-1} W_{hr} + b_{r}) \end{aligned}$$

2 $$\begin{aligned} z_{t}= & {} \sigma (W_{z} \cdot [h_{t-1},x_{t}]) = \sigma (x_{t} W_{xz} + h_{t-1} W_{hz} + b_{z}) \end{aligned}$$

3 $$\begin{aligned} \bar{h}_{t}= & {} tanh (W \cdot [r_{t} * h_{t-1},x_{t}]) = tanh (x_{t} W_{xh} + r_{t} * h_{t-1} W_{hh} + b_{h}) \end{aligned}$$

4 $$\begin{aligned} h_{t}= & {} (1-z_{t}) * h_{t-1} + z_{t} * \bar{h}_{t} \end{aligned}$$

Suppose the shape of $x_{t}$ is: ($batch\_size$, $time\_step$, $input\_dim$), which respectively represent a batch of samples input at the same time, the maximum step length of the input sequence, and the dimension of each sequence. In this paper, these three values are 64, 10, and 16,897, respectively, where 16,897 = 64 $\times$ 256 + 513 (see Fig. 2 and Table 1). The shape of $W_{xr}$ is: ($input\_dim$, $num\_hiddens$), where $num\_hidden$ represents the number of units in the hidden layer, which is 1024 in this paper (see Table 1). The shape of $h_{t}$ is: ($batch\_size$, $time\_step$, $num\_hiddens$), and the shape of $W_{hr}$ is: ($num\_hiddens$, $num\_hiddens$), the shape of $b\_r$ is: ($num\_hiddens$). Then we can eliminate the same dimension value through the dot product operation. In the same way, $W_{r}$ and *W* also correspond to the same operation.

For the output of the last layer of the network, the shape of $W_{ho}$ is: ($num\_hidden$, $num\_outputs$), the shape of $b_{o}$ is ($num\_ouputs$), and the value of $num\_outputs$ in this paper is 513. It can be seen that the shape of the input spectrogram of the entire separation network are: (513, 10), which respectively represent the height and width of the spectrogram. The dimensions of the output spectrogram of the entire separation network are: two spectrograms with shape (513, 10), which represent the predicted singing voice spectrogram and the predicted accompaniment spectrogram after separation. Since this paper uses a standard data set, which is supervised learning, in the loss function (Eqs. 8, 9), we can use the two output spectrograms (think of it as two two-dimensional arrays or matrices), with their respective corresponding ground truth singing voice/accompaniment spectrogram for mean square error operation.

5 $$\begin{aligned} o_{t} = \sigma (W_{o} \cdot h_{t}) = \sigma (h_{t} W_{ho} + b_{o} ). \end{aligned}$$

## Experimental setting

### Dataset

We use the MIR-1K dataset^35^, which includes 1000 pieces of 4–13 seconds of speech data. The clips have been extracted from 110 Chinese songs, sung by men and women. For fair comparison, we use the same specific male and specific female (Abjones and Amy) as in^15^ as the training set, containing a total of 175 clips. The remaining 825 clips are used as the test set. The sampling rate is 16000 Hz, and the sampling points are 16 bits. All subsequent experiments use the MIR-1K as dataset. All experiments use the same 175 clips as the training set and 825 clips as the test set.

### Time-frequency masking

Our network does not change the phase of the original speech signal; we combine the phase with the estimated magnitude spectrogram, and then obtain the signal of the predicted source by Inverse Short-Time Fourier Transform (ISTFT). The magnitude spectrogram of the separated singing voice and accompaniment is obtained by time-frequency mask^36,37^. The function of the time-frequency mask^38^ is to supplement the constraint, which makes the sum of the predictions equal to the original mixture signal^7,15^, thereby avoiding unnecessary loss of information:

6 $$\begin{aligned} \bar{o}{_{1t}}= & {} \frac{ \vert \hat{o}{_{1t} (f)\vert }}{\vert \hat{o}{_{1t} (f)} \vert + \vert \hat{o}{_{2t} (f) \vert }} \odot m_t \end{aligned}$$

7 $$\begin{aligned} \bar{o}{_{2t}}= & {} \frac{ \vert \hat{o}{_{2t} (f)\vert }}{\vert \hat{o}{_{1t} (f)} \vert + \vert \hat{o}{_{2t} (f) \vert }} \odot m_t, \end{aligned}$$

where $\odot$ is defined as the element multiplication of the matrix. Assume that the subscript 1 represents the singing voice, and the subscript 2 represents the accompaniment, then $\hat{o}{_{1t}}$ and $\hat{o}{_{2t}}$, respectively represent the output predictions of the last layer of RNN. However, the predictions of not passing time-frequency masking or other similar processing may not be smooth, because ignoring the additional constraints may cause information loss. $\bar{o}{_{1t}}$ and $\bar{o}{_{2t}}$ represent the smooth prediction after the time-frequency mask. $m_t$ is the magnitude spectrogram of the original mixture signal^7^. It can be seen from Eqs. (6, 7) that the time-frequency mask is essential to calculate the proportion of the singing voice and accompaniment in the magnitude spectrogram of the original mixture signal. $\frac{ \vert \hat{o}{_{1t} (f)\vert }}{\vert \hat{o}{_{1t} (f)} \vert + \vert \hat{o}{_{2t} (f) \vert }}$ and $\frac{ \vert \hat{o}{_{2t} (f)\vert }}{\vert \hat{o}{_{1t} (f)} \vert + \vert \hat{o}{_{2t} (f) \vert }}$ are called soft time-frequency masking. Therefore, in the calculation of the loss function in Eqs. (8) and (9), the magnitude spectrogram after the time-frequency mask was actually used as ($\bar{o}{_{1t}}$ and $\bar{o}{_{2t}}$).

### Loss function

Following Huang et al.^15^, we compare two kinds of loss functions to test the effects of separation result: the mean square error (MSE) (Eq. 8) and the combination of the mean square error and the source-interference ratio (MSE-discrim) (Eq. 9). The MSE loss function ($J_{MSE}$) is a conventional loss function which consists of the sum of two square terms. Regarding the spectrogram as a two-dimensional array or matrix, the first item is to calculate the square of the difference between the predicted singing voice spectrogram and the ground truth singing voice spectrogram, and the second item is to calculate the square of the difference between the two accompaniment spectrograms. So reducing the value of the loss function means that our predicted singing voice signal and accompaniment signal are closer to the ground truth singing voice signal and accompaniment signal:

8 $$\begin{aligned} J_{MSE} = { \vert \vert { \bar{o}{_{1t}} - {o}{_{1t}} }\vert \vert }_2^2 + { \vert \vert { \bar{o}{_{2t}} - {o}{_{2t}} }\vert \vert }_2^2. \end{aligned}$$

The MSE-discrim loss function has been improved on Eq. (8), it adds additional constraints which are intended to make the predicted singing voice spectrogram contain less accompaniment part, and make the predicted accompaniment spectrogram contain less singing voice part:

9 $$\begin{aligned} J_{MSE-discrim} = { \vert \vert { \bar{o}{_{1t}} - {o}{_{1t}} }\vert \vert }_2^2 + { \vert \vert { \bar{o}{_{2t}} - {o}{_{2t}} }\vert \vert }_2^2 - \gamma \ { \vert \vert { \bar{o}{_{1t}} - {o}{_{2t}} }\vert \vert }_2^2 - \gamma \ { \vert \vert { \bar{o}{_{2t}} - {o}{_{1t}} }\vert \vert }_2^2 , 0<\gamma <1. \end{aligned}$$

### Separation indicators

We use the bss_eval_sources in the mir_eval package as the indicators for evaluating separation performance. As Eq. (10), the core idea of the evaluation indicators^39^ is to decompose the predicted signal $o_{t}$ into three parts: $e_{target}(t)$, $e_{interf}(t)$ and $e_{artif}(t)$ (since MIR-1K is a standard dataset, it does not contain noise):

10 $$\begin{aligned} \bar{o}{_{t}} = e_{target}(t) + e_{interf}(t) + e_{artif}(t). \end{aligned}$$

As we calculate the three indicators of singing voice, $e_{target}(t)$ represents the part of the target signal (singing voice signal) in the predicted signal, $e_{interf}(t)$ represents the part of the interference signal (accompaniment signal) in the predicted signal, and $e_{artif}(t)$ represents the remaining part after removing $e_{target}(t)$ and $e_{interf}(t)$. It is the noise introduced by the separation algorithm.

Through the above decomposition method, the separation evaluation indicator can be defined as:

11 $$\begin{aligned} SDR= & {} 10log_{10} ( \frac{\vert \vert e_{target} \vert \vert ^2}{\vert \vert e_{interf} + e_{artif} \vert \vert ^2}), \end{aligned}$$

12 $$\begin{aligned} SIR= & {} 10log_{10} ( \frac{\vert \vert e_{target} \vert \vert ^2}{\vert \vert e_{interf} \vert \vert ^2}), \end{aligned}$$

13 $$\begin{aligned} SAR= & {} 10log_{10} ( \frac{\vert \vert e_{target} + e_{interf} \vert \vert ^2}{\vert \vert e_{artif} \vert \vert ^2}). \end{aligned}$$

These three indicators are the most commonly used indicators for evaluating blind source signal separation (BSS). Experiments^39^ have shown that the evaluation indicators have a good correlation with human perception. SIR reflects the ability of the separation algorithm to suppress interference signals, SAR reflects the ability of the separation algorithm to suppress the introduced noise, and SDR reflects the overall separation performance and it is the most important indicator. The units of SDR, SIR and SAR are all measured in decibels (dB). The larger the value of SDR, SIR, and SAR, the higher the separation performance. The purpose of this study is to improve the SDR of the separated singing voice.

Considering that the initial SDR of the mixture signals are different, in order to compare the separation performance more fairly^40^, further defined the normalised SDR (NSDR):

14 $$\begin{aligned} NSDR(T_e,T_o,T_m) = SDR(T_e,T_o) - SDR(T_m,T_o), \end{aligned}$$

where, $T_e$ is defined as singing-voice or accompaniment estimated by the algorithm, $T_o$ is pure singing-voice or accompaniment in the original signal, and $T_m$ is original mixture signal.

Considering that there are multiple songs of different time lengths in the dataset, global NSDR (GNSDR), global SIR (GSIR), and global SAR (GSAR) are defined to measure the separation performance of our method on the entire dataset.

15 $$\begin{aligned} GNSDR= & {} \frac{ \sum _{i} \alpha _{i} \, NSDR_{i}}{\sum _{i} \alpha _{i}}, \end{aligned}$$

16 $$\begin{aligned} GSIR= & {} \frac{ \sum _{i} \alpha _{i} \, SIR_{i}}{\sum _{i} \alpha _{i}}, \end{aligned}$$

17 $$\begin{aligned} GSAR= & {} \frac{ \sum _{i} \alpha _{i} \, SAR_{i}}{\sum _{i} \alpha _{i}}, \end{aligned}$$

where $\alpha _{i}$ represents the length of the *i*th song. Thus, the final separation indicators in this paper are GNSDR, GSIR and GSAR.

### Perceptual evaluation of speech quality

Perceptual evaluation of speech quality (PSEQ)^41^ is an objective evaluation method to evaluate the effect of subjective audition of speech; it is used to calculate the Mean Opinion Score—Listening Quality Objective (MOS-LQO) value of the speech signal. PESQ compares the output signal extracted when the signal is transmitted through the device with the reference signal, and calculates the difference value between the two. Generally used to evaluate the quality of speech services and the effect of speech enhancement, such as speech quality during telephone calls and denoising quality. In general, the greater the difference between the output signal and the reference signal, the lower the calculated PSEQ and MOS-LQO parameter values. In this paper, we introduce PESQ as another indicator to evaluate the separation effect.

**Figure 5:**
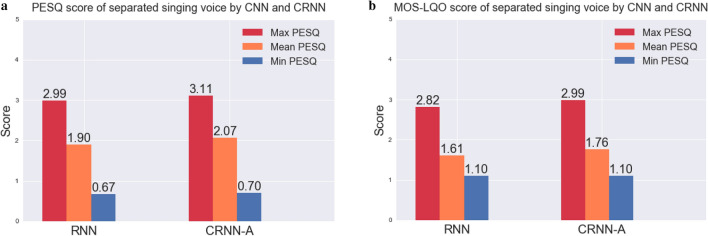
The PESQ and MOS-LQO values on the test set of the MIR-1K dataset under the RNN and our CRNN-A.

The original PESQ score provided by ITU-T P.862 ranges from − 0.5 to 4.5 points. In order to obtain a score that can be compared with the MOS score, we also map this original score to MOS-LQO. The larger the two values are, the better the effect. We take the ground truth singing voice signal as the reference signal, and the singing voice signal predicted by CRNN-A (6 convolutional layers, reduction ratio 16) as the output signal, and calculate the PESQ between the two and its corresponding MOS-LQO value on the entire test set. As shown in Fig. 5, our CRNN-A obtained a gain of 0.17 compared to RNN on the mean PESQ score of the separated singing voice, and achieved a gain of 0.15 on the mean MOS-LQO score.

### Parameters setup

The parameters of the entire network are shown in the Table 1.

## Results

### Experiments with different loss functions (CRNN)

We first use two loss functions given by Eqs. (8) and (9) to compare the separation effect. Since the extra constraint term of the MSE-discrim loss function contains the hyperparameter $\gamma$, in order to facilitate discussion and comparison, we must first fix its value. In order to only compare the effects of different loss functions on separation, the neural network we used in this part does not contain an attention mechanism (i.e. CRNN). The influence of parameter *γ* on separation performance are shown in Table 2.

**Table 2:** The influence of parameter $\gamma$ on separation performance.

Singing-voice				Accompaniment		
$\gamma$	GNSDR	GSIR	GSAR	GNSDR	GSIR	GSAR
$\gamma = 0.001$	7.79	13.29	10.29	7.13	9.68	11.84
$\gamma = 0.01$	7.75	13.66	10.01	7.04	9.67	11.70
$\gamma = 0.05$	7.68	14.61	9.58	7.02	9.94	11.35
$\gamma = 0.5$	6.57	16.39	7.79	6.12	9.75	9.95

**Figure 6:**
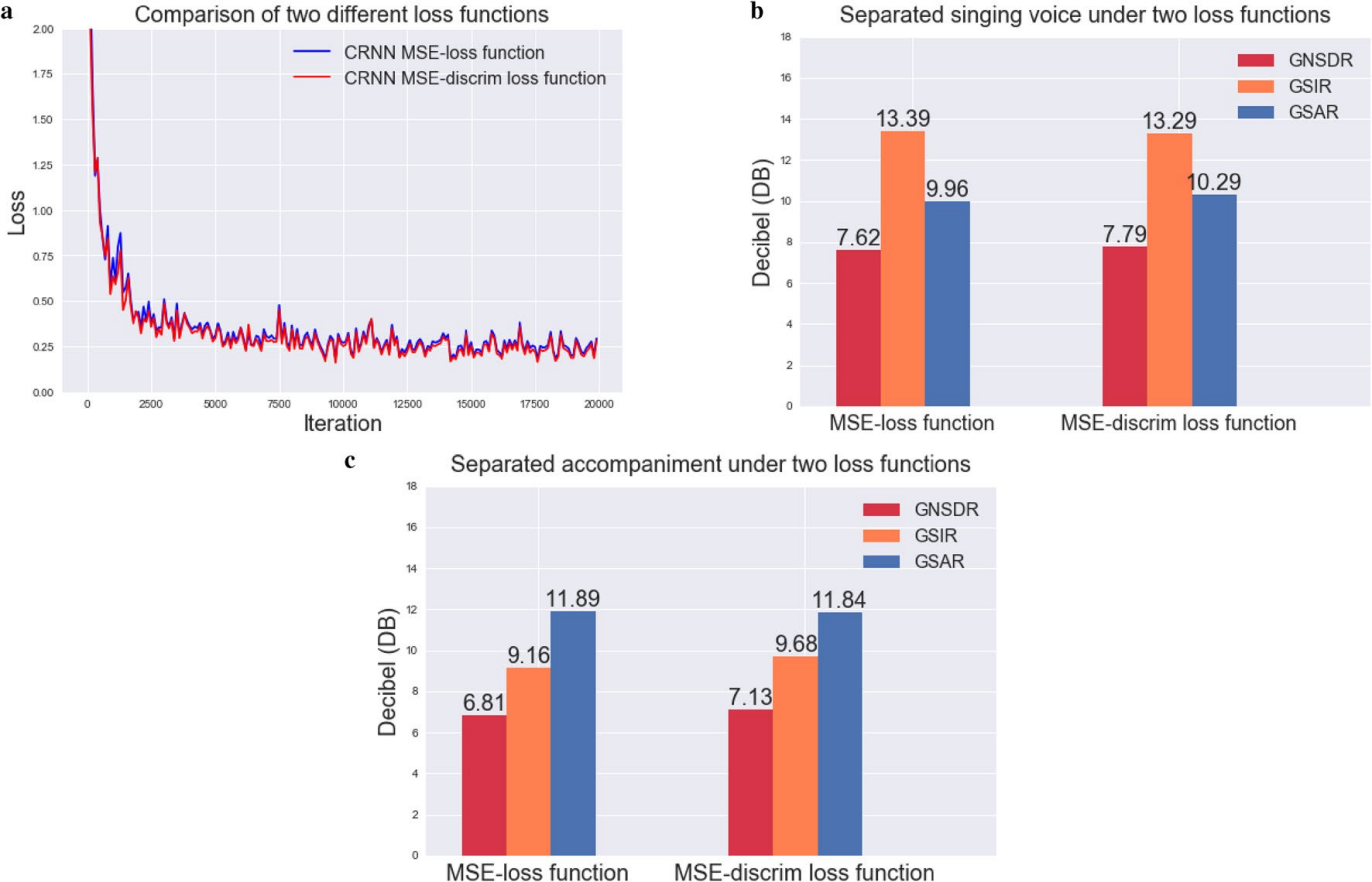
The influence of loss function on separation performance.

It can be seen from Table 2 that as $\gamma$ increases, the value of GSIR increases, while the values of GNSDR and GSAR decrease, indicating that the separation algorithm’s ability to suppress interference signals is enhanced; thereby reducing the part of the interference signal in the predicted signal. It can be seen from the definition of SIR in Eq. (12) that $e_{interf}$ indeed decreases, and the reduction of $e_{interf}$ is greater than $e_{target}$. However, GNSDR is the most important indicator for evaluating separation performance. In order to improve GNSDR, we will fix $\gamma$ to 0.001, and improve the three indicators namely, GNSDR, GSIR, and GSAR by adding an attention mechanism in the follow-up experiments. The comparison results of the two different loss functions are shown in Fig. 6. We see that the MSE-discrim loss function ($\gamma$ = 0.001) is significantly better than the MSE loss function; thus, we choose the final separated loss function as Eq. (18), and the loss functions of subsequent experiments are all Eq. (18).

18 $$\begin{aligned} J_{MSE-discrim} = { \vert \vert { \hat{o}{_{1t}} - {o}{_{1t}} }\vert \vert }_2^2 + { \vert \vert { \hat{o}{_{2t}} - {o}{_{2t}} }\vert \vert }_2^2 - 0.001 (\ { \vert \vert { \hat{o}{_{1t}} - {o}{_{2t}} }\vert \vert }_2^2 + \ { \vert \vert { \hat{o}{_{2t}} - {o}{_{1t}} }\vert \vert }_2^2). \end{aligned}$$

### Experiments with attention mechanism (CRNN-A)

On the basis of the above CRNN, we further added the attention mechanism (i.e. CRNN-A). In view of the effect of the hyperparameter (reduction rate r) in the attention mechanism on the separation performance, a set of experiments were done, as shown in Table 3, where, the “original” in the first row of Table 3 represents the result without the attention mechanism (ie. CRNN), which corresponds to the result of the first row of Table 2.

**Table 3:** The influence of reduction ratio r on separation performance.

Singing voice				Accompaniment		
Reduction ratio r	GNSDR	GSIR	GSAR	GNSDR	GSIR	GSAR
Original	7.79	13.29	10.29	7.13	9.68	11.84
4	7.81	13.48	10.22	7.18	9.87	11.71
8	7.89	13.75	10.17	7.12	9.62	11.97
16	7.86	13.45	10.28	7.23	9.88	11.79
32	7.80	13.46	10.19	7.17	9.91	11.65

From Table 3, we found that the values of GNSDR and GSIR have increased, and the value of GSAR has decreased a little. Analysing the results, we find that its response is in line with the actual logic. According to the meaning of each indicator, a decrease in GSAR means that the separation algorithm’s ability to suppress the introduced noise is weakened, that is, the noise ($e_{artif}$) introduced by the separation algorithm increases. The increase of GNSDR means that the overall separation effect is better, that is, the part of the target signal in the predicted signal ($e_{target}$) has increased. And the increase of GSIR means that the separation algorithm’s ability to suppress interference signals is also enhanced, that is, the part of the interference signal in the predicted signal ($e_{interf}$) has decreased. According to Eq. (10), the sum of the three is determined, indicating that the sum of the rising amplitude of $e_{artif}$ and $e_{target}$ is the same as the falling amplitude of $e_{interf}$, which conforms to the actual law. Thus, we sacrificed the GSAR indicator in exchange for the increase in the primary target GNSDR. Similar conclusions can be obtained in Table 5 of the subsequent experiment. In Table 5, we also compensate for the loss of GSAR by deepening the number of network layers.

**Figure 7:**
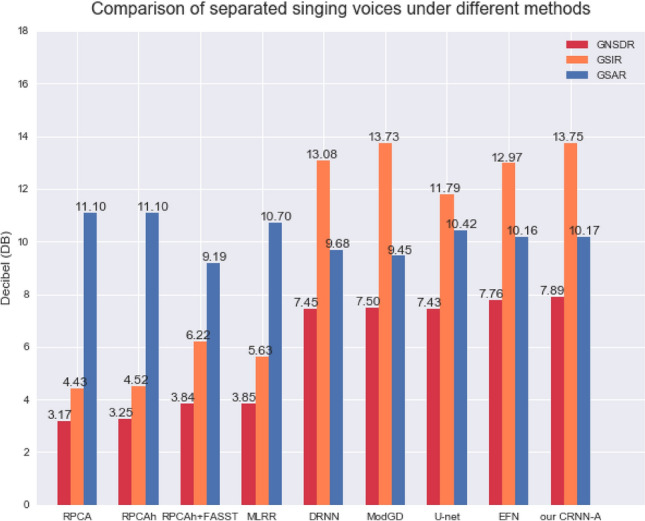
Comparison of singing voice separation under different methods.

Thus, the comparison between our CRNN-A (where the reduction ratio is 8) and other algorithms for the separated singing voice is as shown in Fig. 7.

It can be seen that our method has already surpassed other methods such as EFN^26^ in terms of GNSDR, GSIR and GSAR. However, it is not as good as U-net^23^ on GNSAR. Although improving GNSDR is our primary goal, we suspect this situation may be due to the fact that our network layers are too shallow, and that the shallow neural network’s ability to learn features and its generalisation ability are relatively poor, which indicates underfitting. Deeper neural networks can improve the learning ability, and make it possible to solve more complicated issues, which is also in line with the development trend of deep learning. Therefore, we have done the following supplementary experiments to prove the effectiveness and reproducibility of our proposed method.

### Experiments with different depth convolutional layers (CRNN)

**Figure 8:**
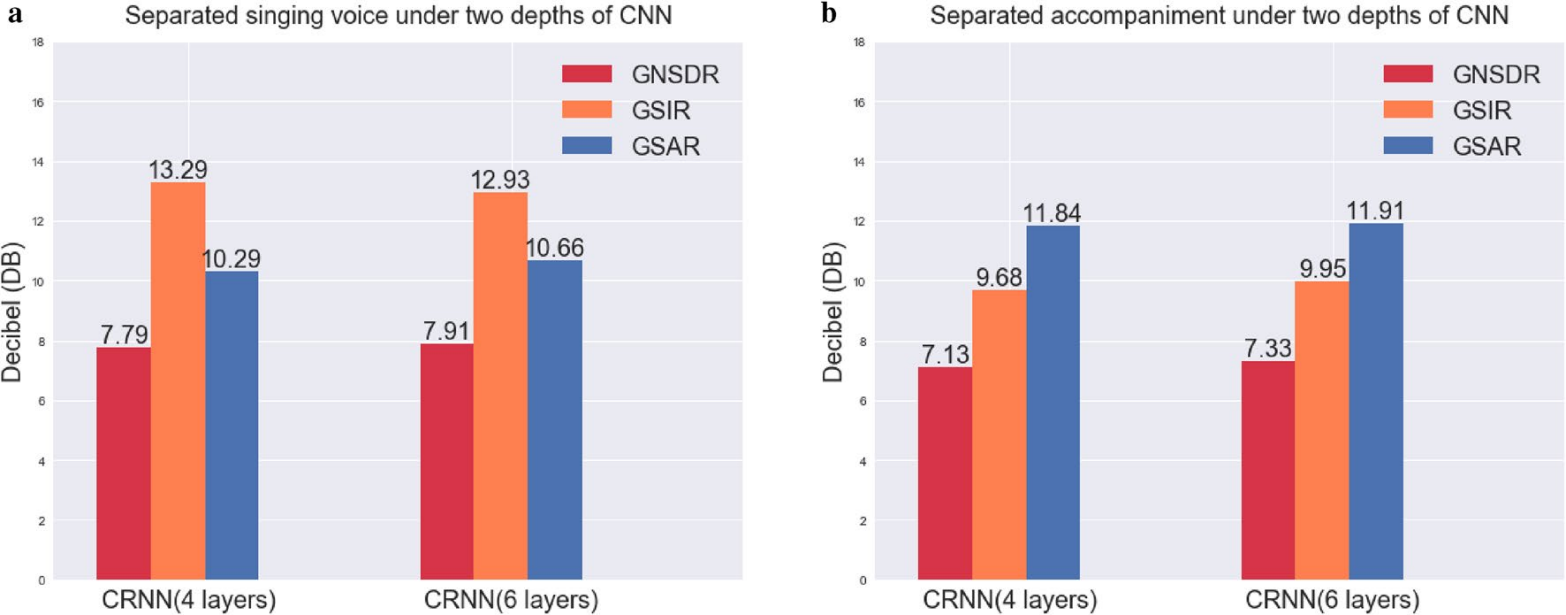
The separation effect of CRNN at different depths, (**a**) is the comparison of the separated singing voice at two depths, (**b**) is the comparison of the separated accompaniment at two depths.

In this paper, we discuss a simple yet effective neural network structure containing only four convolutional layers and three layers of RNN. In order to prove the effectiveness and reproducibility of the CRNN framework, we continue to further deepen the above network. After the last convolutional layer in Fig. 2, two more convolutional layers are added, and the number of output feature maps are 80 and 128, respectively. We first compare CRNN under two different deep networks (ie. neither one adds an attention mechanism), and the effects of separated singing voice and accompaniment are shown in Fig. 8. Although the GSIR of the singing voice has been reduced a little, this is acceptable because our primary goal is to improve GNSDR. This is also in line with the actual error law, we cannot guarantee that all indicators will be improved at all times. From Fig. 8, we can see that the other five indicators have increased, which proves the rationality of selecting CNN as the front-end supplement of RNN and realises the advantages of deep learning.

### Experiments for deeper convolutional layers with attention mechanism (CRNN-A)

Based on the above CRNN with 6 convolutional layers, we continue to add attention mechanisms with different reduction ratios to verify the effectiveness of our CRNN-A framework. The results are shown in Table 4, where, the “original” in the first row of Table 4 represents the result without the attention mechanism, corresponding to the CRNN result of Fig. 8. It can be seen from Table 4 that in a deeper network, our proposed CRNN-A achieves a further improvement in separation performance by adding an attention mechanism, and the law conforms to Table 3.

**Table 4:** The influence of reduction ratio r on separation performance after the network layer is deepened.

Singing voice				Accompaniment		
Reduction ratio r	GNSDR	GSIR	GSAR	GNSDR	GSIR	GSAR
Original	7.91	12.93	10.66	7.33	9.95	11.91
4	7.95	13.17	10.58	7.41	10.13	11.85
8	7.97	13.50	10.45	7.24	9.77	12.00
16	8.07	13.64	10.49	7.34	9.90	12.07
32	7.96	13.33	10.54	7.32	9.94	11.95

Thus, the final comparison between our CRNN-A (6 convolutional layers, reduction ratio 16) and other algorithms for the separated singing voice is shown in Table 5. It can be seen that our method gave good results for every separation indicator.

**Table 5:** The comparison of separated singing voices under different methods after the network layer is deepened.

Singing voice			
Method	GNSDR	GSIR	GSAR
RPCA($\lambda$ = $\lambda _0$)^7^	3.17	4.43	11.10
RPCAh($\lambda = \lambda _0$)^8^	3.25	4.52	11.10
RPCAh $+$ FASST^8^	3.84	6.22	9.19
MLRR^9^	3.85	5.63	10.70
DRNN^15^	7.45	13.08	9.68
ModGD^17^	7.50	13.73	9.45
U-net^23^	7.43	11.79	10.42
EFN^26^	7.76	12.97	10.16
Our CRNN-A	8.07	13.64	10.49

### Mel spectrogram

**Figure 9:**
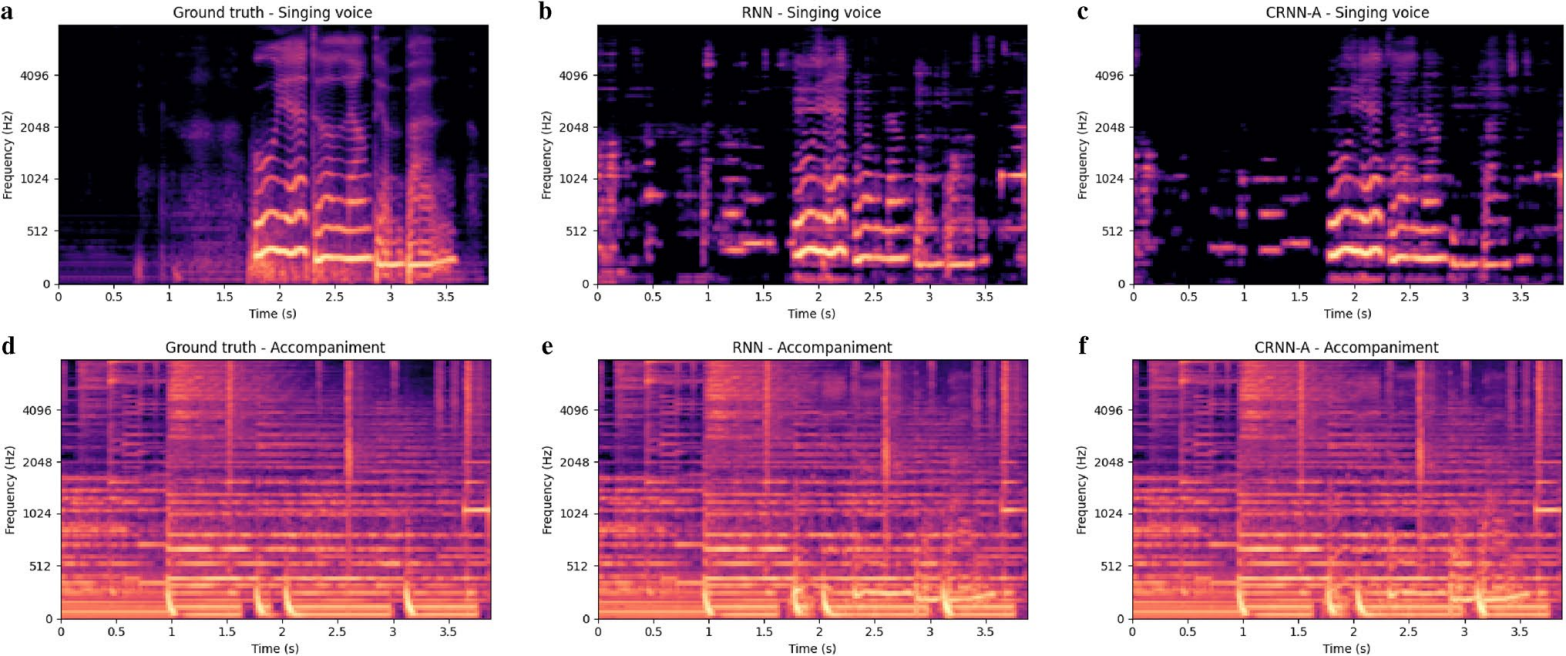
Taking the annar_3_05.wav signal in MIR-1K dataset as an example, the horizontal axis represents time and the vertical axis represents frequency. (**a**) is the ground truth singing voice signal, (**d**) is the ground truth accompaniment. (**b**) and (**e**) are respectively the singing voice signal and accompaniment signal predicted by RNN. (**c**) and (**f**) are respectively the singing voice signal and the accompaniment signal predicted by our CRNN-A.

**Figure 10:**
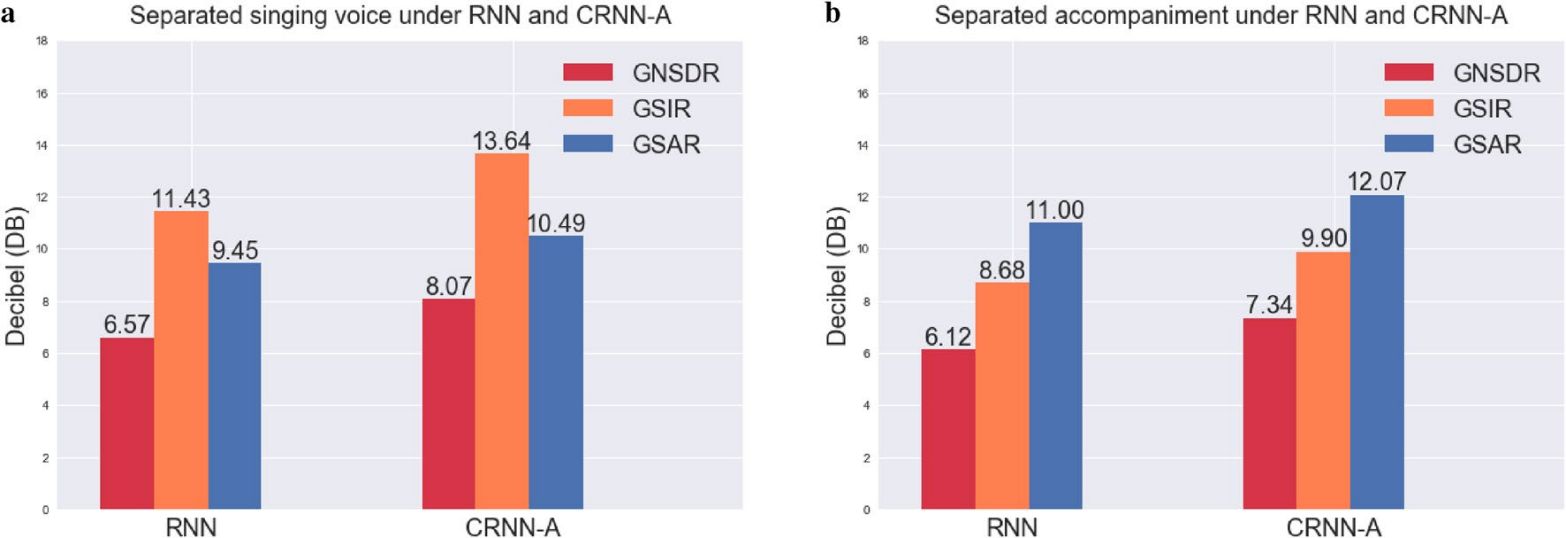
The GNSDR, GSIR and GSAR values on the test set of the MIR-1K dataset under the RNN and our CRNN-A.

In order to more intuitively compare the performance improvement of our method relative to the baseline RNN, we compare the Mel spectrograms generated by our method and the baseline RNN. Mel spectrogram comparison of our CRNN-A (6 convolutional layers, reduction ratio 16) and baseline RNN for singing voice and accompaniments are shown as Fig. 9. It can be seen that compared to our method, the baseline RNN has more artefacts at 1024–4096 Hz in the time period of 0–1.5. In the time period after 3.5, our method also produces fewer artefacts than RNN. In the frequency range of 2048–4096 Hz around the time period 2.5, our method better captures the harmonic signal. Figure 10 shows the different separation indicators between RNN and CRNN-A in decibels.

## Discussion

In this section, we analyse the creativity of the proposed method from different perspectives.

### Combination of CNN and RNN

DRNN^15^ directly takes the original spectrogram as input for separation, which proves that RNN has strong separation ability. On this basis, we have designed CNN as a front-end supplement to RNN based on the following three aspects:

- *Multi-level feature extraction* Different levels of convolution operations can extract different features of the input spectrogram: the shallow convolution layer may only extract some basic features (such as the overall structural features of the spectrogram), and deep convolution operations can obtain fine details (such as the harmonic information of the spectrogram). Therefore, the entire front-end CNN can provide the back-end RNN with richer, multi-level feature information.
- *A special variant of the ResNet*^43^ Inspired by ResNet, we connect the original spectrogram to the last layer (pooling layer) of the front-end; the original spectrogram can be seen as a special shortcut-connection channel in ResNet, which can utilise deeper network models to learn feature information.
- *Feature fusion* Because the features extracted by the front-end CNN and the original spectrogram have different resolutions, concatenating the two can be regarded as a fusion of features. On the other hand, it can also be seen as a connection between the global and local features. The function of the low-resolution feature map is to extract the context information in the spectrogram, and the function of the high-resolution feature map is to restore the fine details of the time-frequency domain^44^. Similarly, T. Sercu et al.^45^ proved that convolution operation along the frequency axis is effective for speech signals. As the convolution kernel slides over different positions, each output after convolution contains specific time-frequency information^28^.

### The attention mechanism

Using CNN as the front-end can provide richer feature information for RNN, but this additional feature information will inevitably cause information redundancy to a certain extent. This is why we incorporate the attention mechanism. The attention mechanism^27^ was originally proposed to improve the recognition accuracy in the field of image detection and classification. Its core idea is to learn the weight corresponding to its importance for each feature map. As shown in the experiment done by Hu et al.^27^, after the attention mechanism is added to ResNet-50, the top-1 and top-5 error rates of image classification are reduced. When we migrate it to NLP and apply it to the task of speech separation, we find that it will also improve the separation performance. As shown in Tables 3 and 4, after adding the attention mechanism on the basis of CRNN, we found that the GNSDR and GSIR of the separated singing voice part have improved, which means that the overall separation effect is improved, and the ability of the separation algorithm to suppress interference signals is enhanced. Although GSAR is reduced, it is still acceptable, because the primary goal of the separation is to increase the overall separation performance (GNSDR). By comparing the networks of different depths (Tables 3 and 4), we see that as the network deepens, the value of GSAR will increase, and the loss of GSAR in Table 3 will be compensated accordingly.

Regarding the CRNN-A framework mentioned in this paper to increase the consistency of the three separation indicators (GNSDR, GSIR, GSAR), our analysis is as follows: Notably, ResNet-50^27^ in the image processing field has 50 hidden layers, yet our CRNN-A in the speech processing field, as detailed in this article, has only four layers (Fig. 7) and six convolutional layers (Table 5). Thus, our network depth is far less than that of a network intended for image processing; meaning our network is simpler yet effective. However, due to the differences in the characteristics of the image and speech signals, it is impossible to obtain satisfactory results for all indicators. However, the CRNN-A framework can improve GNSDR to a certain extent, that is, improve the overall separation effect, and the experimental results shown in Table 5 show that our method comprehensively surpasses other separation methods.

### Reduction ratio r

From Tables 3 and 4, we can see that choosing different reduction ratios r can improve the separation performance to a certain extent. However, the larger the reduction ratio (r = 32) or the smaller (r = 4) does not make the separation effect optimal. The optimal separation effect is often between the two. We analyze the reasons as follows:

The function of the reduction ratio r is to make the network adaptively learn the importance of each channel through dimensionality reduction and dimensionality upgrade operations (Fig. 3), which can be regarded as a special encoding and decoding. In the paper *Squeeze-and-Excitation Networks*, the author Hu Jie on the ImageNet dataset in the field of image classification, through experiments with different reduction ratio r, it is found that the smaller (r = 4) or larger (r = 32) reduction rate cannot make the evaluation of the image classification effect best (ie. top-1 error and top-5 error cannot achieve the smallest). Hu’s experimental conclusions in the image field are consistent with the speech separation experimental conclusions of this paper (Tables 3 and 4).

On the other hand, the smaller the reduction ratio, the greater the complexity of the model (as shown in Fig. 3, r is used as the denominator, if r is smaller, then (64/r), that is, the greater the number of output channels, it will increase the overall network complexity). Therefore, in order to balance the relationship between the complexity of the entire network and the separation performance, we recommend selecting a reduction ratio r of 8 or a reduction ratio r of 16, so that the attention mechanism can maximize the calibration ability of the importance of each channel as well as improve the separation performance.

## Conclusion

In this paper, we have proposed a CRNN-A framework to conduct speech separation studies. The results show that our method exceeds baseline RNN and other separation methods. The core idea of this paper is to effectively combine CNN, which has advantages in image processing, and RNN, which has advantages in processing speech signals. We use the front-end to exploit richer feature information of the spectrogram, and further focus on the corresponding weight distribution for different feature maps. A series of experiments show the effectiveness of our framework, which will also provide new ideas for other tasks in speech processing. In the future, the separation study can further improve the performance by designing more complex front-end structures, data enhancement, and modelling of different frequency bands.
